# A Case of Hydrophobia; with the Appearances on Dissection

**Published:** 1791

**Authors:** John Ferriar

**Affiliations:** Physician to the Infirmary at Manchester.


### MEDICAL FACTS

AND

### OBSERVATIONS.

I. *Case of Hydrophobia; with the Appearances on Diſſection. Communicated in a Letter to* Samuel Foart Simmons, *M. D. F. R. S. by* John Ferriar, *M. D. Phyſician to the Infirmary at Mancheſter.*

ON Friday morning, December 3, 1790, I was deſired to viſit John Johnſon, recommended as a home patient of the Infirmary, who was ſaid to have been bitten by a mad dog.

I found him in a tremulous, irritable ſtate, with a weak, irregular pulſe, and a white tongue. His eyes looked wildly; he was fearful of every unexpected noiſe, and ſeemed to be continually on the watch againſt ſurpriſes. When interrogated reſpecting his complaints, he gave a long detail of pains in his cheſt, cough, and difficulty of breathing; but was unwilling to mention his dread of water. He owned that, a conſiderable time before, he had been bitten in the left cheek by a ſtrange dog, which leaped at his face in paſſing while he was at work in the ſtreet. The accident affected him ſo little, that the preciſe date of it had eſcaped his memory. He gueſſed it to have happened more than three months ago. Since that time he had been twice afflicted with complaints ſuppoſed to be pleuritic, which were removed by bleeding, bliſtering, and other remedies, of which he could give no account. He had been bled twice within the laſt week, and had a bliſter on the left ſide of the cheſt when I ſaw him.

On Monday, November the 29th, in the evening, his wife had obſerved, for the firſt time, that he ſwallowed ſome gin and water with reluctance and difficulty; the uneaſineſs in ſwallowing liquids ſoon became his principal complaint, but the bite was not recollected till Thurſday evening; when a medical gentleman, who was applied to, inquired whether he had ever been bitten by a dog. Even then he recollected the circumſtances but imperfectly. He got got down folids with great eafe during the whole complaint.

When I defired him to drink a little water, he fhewed ftrong marks of difguft, but, recollecting himfelf, faid he would try; that he did not believe the dog to have been mad, (an idea in which I encouraged him), and that he was not afraid of water. As foon as he touched the cup, I perceived fome fpafmodic contractions of the mufcles of deglutition, and when he raifed it towards his mouth the mufcles of the cheeks were ftrongly contracted, and a fort of convulfive gulping was very frequently repeated. After one or two unfuccefsful attempts, he fwallowed a fmall quantity of water, but with a violent ftruggle, fucceeded by univerfal tenfion; and he would not be perfuaded to make another trial.

He complained that cold air affected his throat with a fimilar uneafinefs; and when afked where the impreffion was felt, pointed to his throat, immediately under the thyroid cartilage. The opening of the door always made him complain.

His difcourfe was fomewhat incoherent, and he frequently referred, with fome degree of terror, to the circumftance of the bite.

By his wife's account, he had been a fober, induftrious man; abftemious with refpect to food; and addicted to no practices likely to pervert his imagination. His age was thirty-nine; his evacuations were in a natural ftate.

The fcar on his cheek, which was between the ear and the angle of the jaw, but rather more advanced, was hardly difcernible: his wife remembered to have feen it bloody. I had him removed to the Hofpital as foon as poffible, that he might enjoy every advantage of attendance; and till I could have the fatif-faction of confulting with my brethren, ordered him to take a bolus, containing a fcruple of bark, fix grains of mufk, and half a grain of opium: he was immerfed in the cold bath, and was directed to fwallow, as often as poffible, a portion of a mixture of vinegar and water.

After his removal, as his wife had informed me that the found of water diftreffed him, I di-rected fome to be poured out in the paffage ad-joining to his room. He ftarted at the noife, looked wildly round, begged to be fent home, and faid he was not afraid of water.

At five o'clock in the afternoon we met in confultation, when the horror of water, and difficulty ficulty of fwallowing it, were afcertained in prefence of all the phyficians to the houfe.

We agreed to fcarify the cicatrix on the cheek deeply, and to apply a blifter over the incifions: a bolus, containing a fcruple of bark, fifteen grains of mufk, and two grains of opium, was directed to be given every four hours; two drachms of ftrong mercurial ointment were applied to the throat, arms, and groins; a mixture of eight ounces of diftilled vinegar, and twelve ounces of decoction of bark, was ordered, of which three or four table-fpoonfuls were to be given as frequently as poffible; and a poultice, confifting of three drachms of galbanum, two fcruples of opium, and one drachm of camphor, was applied, after the mercurial friction, to the throat.

About nine o'clock the fame evening I faw him again. He had fwallowed his medicines without much reluctance, but was more incoherent, and complained greatly of cold.

During the night his delirium increafed: he was very reftlefs, impatient, and intractable. He threw himfelf out of bed repeatedly, and with his nails fcratched the hand of one of the keepers who attempted to replace him. However, he took four bolufes, and fwallowed more

 than than a pint of his mixture. He had one ſtool before morning.

At nine o'clock on Saturday morning, December 4th, we met again in conſultation. We found that his difficulty in ſwallowing liquids was leſs: he had taken ſome very thin porridge, the uſual breakfaſt of the houſe; and he drank ſeveral draughts of his mixture, without any ſtriking appearance of diſguſt, in our preſence; but his eyes were heavy, and inclined to fix, his pulſe much ſunk, and there was a conſtant tendency to low delirium. We, therefore, concluded that the termination of the diſeaſe approached; but directed that the plan we had agreed on ſhould be purſued as long as he ſhould be capable of ſwallowing. Before I left him he retched ſeveral times, and brought off ſome wind: half a grain of emetic tartar was directed to be added to his next bolus, but he did not live to take it. At a quarter paſt ten he ſwallowed ſome of his mixture, and immediately after threw up a part of it again. He then fell into convulſions, and died in the courſe of a few minutes.

I was very deſirous of examining the body as early as poſſible, that the appearances attending this dreadful diſorder might be fairly aſcertained; tained ; the inflammation of the ſtomach, deſcribed in former diſſections, having been often attributed to the action of the gaſtric juice. Accordingly, the body was opened by Mr. Simmons, at a quarter before three o'clock on Saturday afternoon, in preſence of moſt of the phyſicians and ſurgeons of the hoſpital.

In the brain, which was the part firſt examined, the only preternatural appearance was a diſtention of the pia mater, on both hemiſpheres, with a limpid fluid. The quantity of water in the lateral ventricles, at the baſis of the brain and round the ſpinal marrow, appeared to be ſomewhat unuſual. The lungs were uncommonly ſound, excepting one ſlight adheſion at the poſterior part of the left lobe. The trachea was perfectly ſound. The pericardium adhered pretty firmly to the heart in its whole compaſs. The ſtomach and inteſtines ſeemed, externally, ſound; but on opening the œſophagus a morbid appearance preſented itſelf. About two inches above the cardia the epidermis of the œſophagus was abraded in irregular points, and expoſed an inflamed ſurface of a dark red colour : ſtill lower, the abraſions became linear, and extended into the ſtomach itſelf. The edges of the epidermis, ſurrounding the abraſions, were unequal, and elevated. A fimilar affection was traced along the leffer curvature of the ftomach, but fainter in its progrefs to the pylorus, where it was leaft difcernible, and about which it feemed to terminate. The whole of the inflamed parts bore a ftriated appearance, darkeft in the œfophagus, and lighteft and more indiftinct towards the pylorus. The ftomach was half full of a dark-coloured fluid, which fmelt ftrongly of mufk. The other vifcera were in a natural ftate.

As no more than four hours and a half elapfed between the patient's death and the diffection, I believe the abrafion obferved may be fairly confidered as the effect of the difeafe, efpecially as the ftomach contained a confiderable quantity of fluid; but although the preternatural irritability, produced by this fpecific inflammation, completely explains the peculiar fenfibility to cold water and cold air, yet the dread of water, though conftituting the diagnofis, can only be regarded as *the fymptom of a fymptom*. The local inflammation, even when confidered in connexion with the flight effufion vifible in the brain, is very inadequate to the explanation of the patient's death.

The relief from the difficulty of ſwallowing liquids, obſervable towards the cloſe of this caſe, is not a ſolitary inſtance; the diſeaſe has even been ſaid to exiſt without any horror of water, or difficulty of receiving it into the ſtomach *.

The eaſe with which ſolids were ſwallowed by our patient admits an obvious explanation. In the irritable ſtate of the œſophagus, the comparatively ſmall degree of contraction neceſſary for the deſcent of ſolid food, is performed without difficulty. For the deglutition of liquids a very ſtrict contraction is required, which ſtrains and irritates the inflamed parts, and conſequently occaſions great diſtreſs.

Little information reſpecting the practice in hydrophobia can be drawn from this caſe; yet if other obſervations ſhould confirm the opinion, that a peculiar inflammation exiſts in the ſtomach and œſophagus, in all inſtances of this diſeaſe, I conceive that ſome meaſures ſhould be taken to counteract it, though it appears to be only a ſymptom of the general diſorder. Bliſters applied to the throat, or between the

* See Mead's works; Lieutaud, Precis de la Med. prat.; and ſome late articles in the newſpapers.

ſhoulders,

ſhoulders, might be uſeful; and if another ſimilar caſe ſhould unhappily occur to me, I ſhould certainly employ them. Our patient had a recent bliſter on his ſide.

Lieutaud* enumerates very extenſive appearances of inflammation and ſuppuration in the ſtomach and bowels of hydrophobic patients diſcovered on diſſection, and other writers have mentioned inflammation of the ſtomach, in ſuch caſes, in general terms; but perhaps ours is the moſt ſatisfactory examination yet obtained, on account of its nearneſs to the death of the patient. Lieutaud mentions the adheſion of the pericardium to the heart, among other appearances.

I ſhould conceive blood-letting to be a very ambiguous remedy in this complaint: with us it was prohibited by the ſtate of the pulſe, the advanced period of the diſeaſe, and the free uſe made of it a few days before by the patient.

The large uſe of mercurial frictions is ſaid to have been ſucceſsful in hydrophobia. It has perhaps been ſuggeſted by the determination to the ſalivary glands, ſo remarkable in the courſe of the diſeaſe. I own I have ſome

* Precis de la Medecine pratique, Tome II. p. 92. 8vo. Paris, 1777.

doubts doubts refpecting the propriety of ufing a remedy which produces fo great a degree of irritability in the ftate of high irritation attending hydrophobia. The appearance of the inflamed parts approaches to the eryfipelatous ftate; and the whole train of fymptoms feems to require the aid of the cold bath, and the free ufe of bark and opium.

*Manchefter,*

December 12, 1790.

